# Clinical and prognostic implications of C‐reactive protein levels in myocardial infarction with nonobstructive coronary arteries

**DOI:** 10.1002/clc.23651

**Published:** 2021-05-25

**Authors:** Kai M Eggers, Tomasz Baron, Marcus Hjort, Anna M Nordenskjöld, Per Tornvall, Bertil Lindahl

**Affiliations:** ^1^ Department of Medical Sciences and Uppsala Clinical Research Center Uppsala University Uppsala Sweden; ^2^ Faculty of Health, Department of Cardiology Örebro University Örebro Sweden; ^3^ Department of Clinical Science and Education, Södersjukhuset Karolinska Institute Stockholm Sweden

**Keywords:** C‐reactive protein, inflammation, MINOCA, myocardial infarction, prognosis

## Abstract

**Background:**

Myocardial infarction with nonobstructive coronary arteries (MINOCA) is a heterogeneous condition. Recent studies suggest that MINOCA patients may have a proinflammatory disposition. The role of inflammation in MINOCA may thus be distinct to myocardial infarction with significant coronary artery disease (MI‐CAD).

**Hypothesis:**

We hypothesized that inflammation reflected by C‐reactive protein (CRP) levels might carry unique clinical information in MINOCA.

**Methods:**

This retrospective registry‐based cohort study (SWEDEHEART) included 9916 patients with MINOCA and 97 970 MI‐CAD patients, used for comparisons. Multivariable‐adjusted regressions were applied to investigate the associations of CRP levels with clinical variables, all‐cause mortality and major cardiovascular events (MACE) during a median follow‐up of up to 5.3 years.

**Results:**

Median admission CRP levels in patients with MINOCA and MI‐CAD were 5.0 (interquartile range 2.0–9.0) mg/dl and 5.0 (interquartile range 2.1–10.0 mg/dl), respectively. CRP levels in MINOCA exhibited independent associations with various cardiovascular risk factors, comorbidities and estimates of myocardial damage. The association of CRP with peripheral artery disease tended to be stronger compared to MI‐CAD. The associations with female sex, renal dysfunction and myocardial damage were stronger in MI‐CAD. CRP independently predicted all‐cause mortality in MINOCA (hazard ratio 1.22 [95% confidence interval 1.17–1.26]), similar to MI‐CAD (p interaction = 0.904). CRP also predicted MACE (hazard ratio 1.08 [95% confidence interval 1.04–1.12]) but this association was weaker compared to MI‐CAD (p interaction<.001).

**Conclusions:**

We found no evidence indicating the presence of a specific inflammatory pattern in acute MINOCA compared to MI‐CAD. However, CRP levels were independently, albeit moderately associated with adverse outcome.

## 1 INTRODUCTION

Myocardial infarction with nonobstructive coronary arteries (MINOCA) is a condition that is gaining increasing interest.^1^, ^2^ The term MINOCA refers to a myocardial infarction (MI) fulfilling the criteria outlined in the Universal Definition^3^ but without coronary stenosis ≥50%. Around 5%–10% of all MI are MINOCA.^1^, ^4^ The etiology of MINOCA is heterogenous with a multitude of causative or contributing mechanisms.^1^ However, recent studies suggest that MINOCA patients as well may be characterized by a proinflammatory disposition in terms of more common inflammatory disorders^5^, ^6^ or as reflected by circulating biomarkers.^7^

Inflammation also represents a key pathobiological mechanism in acute coronary syndromes. C‐reactive protein (CRP), a sensitive downstream marker of the inflammation cascade, has been linked in various studies to adverse outcome in this condition.^8^, ^9^ Given the considerations above, we hypothesized that CRP levels might provide clinical and prognostic information that is unique in MINOCA and at difference to myocardial infarction with significant coronary artery disease (MI‐CAD).

The aims of the present study were to closer investigate the associations of CRP levels in MINOCA with clinical findings and outcome overall, and in comparison to CRP levels in MI‐CAD. For this purpose, a large cohort of Swedish MI patients with data available in the SWEDEHEART (Swedish Web‐system for Enhancement and Development of Evidence‐based care in Heart disease Evaluated According to Recommended Therapies) registry was assessed.

## 2 METHODS

### 2.1 Study population

This study is part of the TOTAL‐AMI (Tailoring Of Treatment in All comers with Acute Myocardial Infarction) project. The primary aim of TOTAL‐AMI is to study the mechanisms and implications of different MI subtypes^3^ and of comorbidities (e.g. chronic obstructive pulmonary disease, atrial fibrillation, renal dysfunction) in MI. TOTAL‐AMI uses data from SWEDEHEART which is a Swedish registry collecting information from consecutive patients admitted to coronary care units or other specialized facilities because of suspected acute coronary syndrome. SWEDEHEART provides almost nationwide coverage and lifelong follow‐up. Upon hospital admission, patients receive written information about the registry, have the right to deny participation and to get their data erased upon request. Written informed consent is not required according to Swedish law.

The population for the present study included all MI patients, admitted between January 2005 and May 2018, with available data for levels of CRP. Only first‐time admissions were considered. To be regarded having MINOCA, MI patients were required to have normal or near‐normal coronary arteries (<50% stenosis), to be free from a history of MI or coronary intervention and not to have undergone a coronary intervention during the index hospitalization. MI patients with significant (≥50%) coronary stenoses were regarded having MI‐CAD. MI‐CAD patients with a history of MI or coronary intervention were excluded from the analyses to avoid the confounding impact of pre‐existing coronary artery disease in comparisons with MINOCA patients.

All data had been made pseudonymized before the statistical analyses. The study was conducted according to the principles of the 1975 Declaration of Helsinki and had been approved by the Regional Ethical Review Board in Stockholm (2012/60–31/2).

### 2.2 Measurement of CRP levels

The CRP levels used in this analysis had been obtained upon admission as part of clinical routine practice in Sweden. Measurements were performed using standard assays at the central laboratories of the respective hospitals participating in SWEDEHEART.

### 2.3 Prognostic evaluation

Information on patient outcome was obtained by merging SWEDEHEART with data from the mandatory Swedish Patient Registry (hospitalization dates and discharge diagnoses based on International Classification of Diseases, 10th revision, Clinical Modification [ICD‐10‐CM] codes) and the Swedish Cause of Death Registry, both held by the Swedish Board of Health and Welfare. The outcomes considered for this analysis were all‐cause mortality with available data until May 2018 and major cardiovascular adverse events (MACE), defined as the composite of cardiovascular mortality, readmissions for recurrent MI (ICD‐10‐CM code I21), hospitalization for heart failure (ICD‐10‐CM code I50) and ischemic stroke (ICD‐10‐CM code I63). During the first 30 days after the index hospitalization, it is not possible to separate a new MI from the index MI in the Patient Registry. Therefore, only MI occurring at least 30 days after the hospitalization were counted to avoid 'contamination' from the index event. Information on MACE was only available until December 2017 since there is a time lag for processing hospitalization data within the Swedish Patient Registry.

### 2.4 Statistical analysis

All continuous variables were skewed and are reported as medians with interquartile ranges (IQR). Differences were tested using the Pearson χ^2^ test. Categorical variables are expressed as frequencies and percentages with differences being assessed using the Kruskal‐Wallis test.

Multiple linear regressions were used to investigate the associations of CRP levels with clinical variables in MINOCA and MI‐CAD, respectively. Model 1 included age, sex, current smoking, hypertension, diabetes mellitus, hyperlipidemia, estimated glomerular filtration rate (Chronic Kidney Disease Epidemiology Collaboration equation), previous heart failure, previous stroke, peripheral artery disease, chronic obstructive pulmonary disease, previous or present cancer, dementia and atrial fibrillation on the admission ECG. In addition, adjustment was made for medications on admission (antiplatelets, betablockers, inhibitors of the renin‐angiotensin‐aldosterone system [angiotensin‐converting‐enzyme inhibitors and angiotensin II receptor blockers] and statins), admission year and hospital. In model 2, previous heart failure was replaced by left‐ventricular ejection fraction estimated by in‐hospital echocardiography. Model 3 was adjusted as model 1 with addition of high‐sensitivity cardiac troponin T (hs‐cTnT) as covariate. Values of CRP and hs‐cTnT were highly skewed and ln‐transformed in alla analyses to achieve normality.

The associations of CRP (ln) with adverse events were studied with the use of Cox regression models. We applied a similar adjustment set as for the multiple linear regressions including ST‐segment changes on the admission ECG but without considering medications upon admission. The prognostic implications of CRP (ln) relative to the MI type (MINOCA vs. MI‐CAD) were assessed by forcing an interaction term CRP (ln)*MI type into model 1 with additional adjustment for in‐hospital coronary revascularization. Prognostic estimates are presented as hazard ratios (HR) with 95% confidence intervals (CI). Cumulative probability curves were constructed using the Kaplan–Meier method, and the log‐rank test was used to compare the occurrence of adverse outcome across CRP quartiles.

In all tests, a two‐sided p value <.05 was considered significant. The software package SPSS 27.0 (SPSS Inc., Chicago, IL) was used for the analyses.

## 3 RESULTS

The MINOCA cohort investigated in the present analysis consisted of 9916 patients. Out of totally 125 713 MI‐CAD patients in the dataset, 97 970 (77.9%) had no history of coronary revascularization or MI and were used for comparative analyses.

The median CRP levels were 5.0 (IQR 2.0–9.0) mg/dl in MINOCA and 5.0 (IQR 2.1–10.0) mg/dl in MI‐CAD. Information on clinical characteristics in MINOCA patients in relation to CRP quartiles is presented in Table 1. Increasing CRP quartiles exhibited significant associations across many cardiovascular risk factors and comorbidities, apart from hyperlipidemia and previous stroke. Increasing CRP quartiles were also associated with more adverse ECG findings, poorer left‐ventricular ejection fraction and higher hs‐cTnT levels. The clinical characteristics of MI‐CAD patients are presented in the Table S1.

**TABLE 1:** Clinical characteristics and outcome in MINOCA patients in relation to CRP quartiles

	CRP (mg/dl)					
	<2.0 (*n* = 2460)	2.0–4.9 (*n* = 2564)	5.0–8.9 (*n* = 2419)	≥9.0 (*n* = 2480)	p value	Missing values
Risk factors						
Age (years)	66 (57–74)	67 (58–74)	67 (58–75)	69 (60–76)	<.001	‐
Males	946 (38.5%)	883 (34.4%)	875 (36.2%)	961 (38.8%)	.004	‐
Current smoking	375 (15.2%)	447 (17.5%)	439 (18.1%)	537 (21.9%)	<.001	7 (0.1%)
Hypertension	1078 (43.2%)	1159 (45.2%)	1079 (44.6%)	1103 (44.5%)	<.001	5 (0.1%)
Diabetes	262 (10.7%)	265 (10.3%)	270 (11.2%)	373 (15.0%)	<.001	‐
Hyperlipidemia	496 (20.2%)	476 (18.6%)	438 (18.1%)	447 (18.0%)	0.188	‐
BMI (kg/m^2^)	25.4 (22.9–28.3)	26.2 (23.8–29.4)	26.4 (23.4–29.8)	26.7 (23.6–30.4)	<.001	1229 (12.4%)
eGFR (ml/min/1.73m^2^)	83.1 (67.5–93.6)	82.4 (67.4–93.1)	82.9 (67.0–93.7)	79.1 (61.5–92.1)	<.001	24 (0.2%)
Medical history						
Heart failure	50 (2.0%)	54 (2.1%)	82 (3.4%)	108 (4.4%)	<.001	1 (0.0%)
Previous stroke	108 (4.4%)	130 (5.1%)	103 (4.3%)	130 (5.3%)	0.234	61 (0.6%)
PAD	30 (1.2%)	42 (1.6%)	50 (2.1%)	83 (3.3%)	<.001	‐
COPD	150 (6.1%)	182 (7.1%)	224 (9.3%)	358 (14.4%)	<.001	‐
Previous/present cancer	42 (1.7%)	47 (1.8%)	55 (2.3%)	82 (3.3%)	.001	‐
Dementia	5 (0.2%)	5 (0.2%)	5 (0.2%)	3 (0.1%)	0.879	‐
ECG findings						
Sinus rhythm	2230 (90.8%)	2292 (89.5%)	2147 (89.0%)	2077 (83.9%)	<.001	20 (0.2%)
Atrial fibrillation	174 (7.1%)	209 (8.2%)	211 (8.7%)	310 (12.5%)	<.001	20 (0.2)%
ST‐elevation	311 (12.7%)	292 (11.4%)	301 (12.5%)	440 (17.8%)	<.001	26 (0.3)%
ST‐depression	359 (14.6%)	403 (15.8%)	465 (19.3%)	432 (17.5%)	<.001	26 (0.3)%
Medications at admission						
Aspirin	431 (17.5%)	474 (18.5%)	436 (18.0%)	529 (21.3%)	.003	‐
P2Y12 blockers	60 (2.4%)	70 (2.7%)	53 (2.2%)	61 (2.5%)	0.679	‐
Oral anticoagulants	100 (4.1%)	142 (5.5%)	131 (5.4%)	178 (7.2%)	<.001	‐
Betablockers	605 (24.6%)	638 (24.9%)	621 (25.7%)	672 (27.1%)	0.178	‐
RAAS‐inhibitors	712 (28.9%)	759 (29.6%)	752 (31.1%)	763 (30.8%)	0.319	‐
CCB	320 (13.0%)	312 (12.2%)	290 (12.0%)	219 (12.9%)	0.625	‐
Statins	485 (19.7%)	464 (18.1%)	418 (17.3%)	433 (17.5%)	0.107	‐
Examination results						
hs‐cTnT (ng/L)^b^	163 (64–415)	168 (62–462)	182 (76–463)	273 (108–628)	<.001	‐
Echocardiographic findings^c^						
LVEF ≥0.50	1643 (80.3%)	1630 (77.7%)	1486 (75.0%)	1291 (63.9%)	<.001	
LVEF 0.40–0.49	238 (11.6%)	267 (12.7%)	293 (14.8%)	355 (17.6%)		
LVEF 0.30–0.39	120 (5.9%)	141 (6.7%)	134 (6.8%)	260 (12.9%)		
LVEF <0.30	44 (2.2%)	61 (2.9%)	68 (3.4%)	114 (5.6%)		
Medications at discharge^d^						
Aspirin	2167 (88.4%)	2218 (86.9%)	2148 (89.2%)	2077 (84.8%)	<.001	‐
P2Y12 blockers	1722 (70.3%)	1730 (67.8%)	1662 (69.0%)	1498 (61.1%)	<.001	‐
Oral anticoagulants	180 (7.3%)	240 (9.4%)	205 (8.5%)	306 (12.5%)	<.001	1 (0.0%)
Betablockers	1927 (78.7%)	1986 (77.9%)	1939 (80.5%)	1995 (81.4%)	.006	‐
RAAS‐inhibitors	1511 (61.7%)	1622 (63.6%)	1609 (66.8%)	1611 (65.8%)	.001	‐
CCB	424 (17.3%)	442 (17.3%)	403 (16.7%)	376 (15.4%)	0.199	1 (0.0%)
Statins	2115 (86.3%)	2165 (84.9%)	2054 (85.3%)	1950 (79.6%)	<.001	‐
Crude event rates^a^						
All‐cause mortality	300 (12.2%)	374 (14.6%)	414 (17.1%)	768 (31.0%)	<.001	‐
CV mortality	98 (4.1%)	151 (6.1%)	140 (5.9%)	249 (10.3%)	<.001	‐
MI	155 (6.4%)	179 (7.2%)	163 (6.9%)	171 (7.1%)	0.751	‐
Heart failure	93 (3.9%)	137 (5.5%)	166 (7.0%)	250 (10.3%)	<.001	‐
Stroke	103 (4.3%)	111 (4.5%)	108 (4.6%)	134 (5.5%)	0.166	‐
MACE	366 (15.2%)	469 (18.8%)	462 (19.6%)	644 (26.6%)	<.001	‐

Abbreviations: BMI, body mass index; CCB, calcium channel blockers; CV, cardiovascular; COPD, chronic obstructive pulmonary disease; eGFR, estimated glomerular filtration rate; RAAS, renin‐angiotensin‐aldosterone system; hs‐cTnT, high‐sensitivity cardiac troponin T; LVEF, left‐ventricular ejection fraction; MI, myocardial infarction; MACE, major cardiovascular adverse events; PAD, peripheral artery disease.

*Note*: Data given as numbers (with percentages) or medians (with interquartile ranges).

^a^ Follow‐up data regarding all‐cause mortality were available in all patients (n = 9916) until May 2018, and regarding MACE and its individual components in 9676 patients until December 2017.

^b^ *n* = 4171.

^c^ Echocardiography was performed in 8268 patients (88.3%). Data on LVEF was available in 8145 of these patients.

^d^ Assessed in in‐hospital survivors: *n* = 9860.

Upon multivariable adjustment, significant and consistent associations were noted between CRP (ln) and current smoking, diabetes mellitus, lower glomerular filtration rate, estimates of myocardial damage (i.e. lower left‐ventricular ejection fraction and higher hs‐cTnT levels), peripheral artery disease, chronic obstructive pulmonary disease, previous or present cancer and atrial fibrillation (Table 2). As illustrated by the non‐overlapping 95% confidence intervals of the regression coefficients in model 2, the association of CRP (ln) with peripheral artery disease tended to be stronger in MINOCA compared to MI‐CAD, and there was a trend towards a stronger association with chronic obstructive pulmonary disease (Table S2B). Among patients with MI‐CAD, CRP (ln) exhibited stronger associations with female sex, lower glomerular filtration rate and depressed left‐ventricular ejection fraction (Table S2B). Even the extent of coronary artery disease (categorized as 1‐ or 2‐vessel disease, 3‐vessel or left main disease, inconclusive angiographic findings) was associated with CRP (ln) in MI‐CAD when added as additional variable to model 2 (β = 0.041; p < .001).

**TABLE 2:** Predictors of CRP levels in patients with MINOCA

	Model 1 (*n* = 9716)		Model 2 (*n* = 8061)		Model 3 (*n* = 4098)	
	β	p value	β	p value	β	p value
Age (10 years)	0.042	.001	0.025	.070	0.072	<.001
Male sex	0.019	.056	0.020	.070	0.009	0.557
Current smoking	0.061	<.001	0.052	<.001	0.067	<.001
Hypertension	−0.001	0.962	0.006	0.655	0.018	0.372
Diabetes	0.061	<.001	0.053	<.001	0.072	<.001
Hyperlipidemia	0.029	0.567	0.037	0.514	0.211	.011
eGFR	−0.075	<.001	−0.053	<.001	−0.033	.070
Heart failure	0.025	.016	‐	‐	0.015	0.347
Previous stroke	0.001	0.928	−0.006	0.615	−0.009	0.560
PAD	0.056	<.001	0.068	<.001	0.069	<.001
COPD	0.103	<.001	0.090	<.001	0.122	<.001
Previous/present cancer	0.030	.003	0.030	.006	0.046	.002
Dementia	−0.010	0.288	−0.002	0.835	−0.014	0.351
Atrial fibrillation	0.042	<.001	0.040	.001	0.043	.009
LVEF	‐	‐	0.115	<.001	‐	‐
hs‐cTnT (ln)	‐	‐	‐	‐	0.111	<.001

Abbreviations: COPD, chronic obstructive pulmonary disease; eGFR, estimated glomerular filtration rate; LVEF, left‐ventricular ejection fraction; hs‐cTnT: high‐sensitivity cardiac troponin T; PAD, peripheral artery disease.

*Note*: Model 1: Analyses were adjusted for all assessed variables, medications at admission (as listed in Table 1), hospital and admission year. Model 2: adjusted as model 1 with replacement of heart failure by left‐ventricular ejection fraction, categorized as ≥0.50, 0.40–0.49, 0.30–0.39 and < 0.30. Model 3: adjusted as model 1 with additional adjustment for high‐sensitivity cardiac troponin T (ln).

**FIGURE 1:**
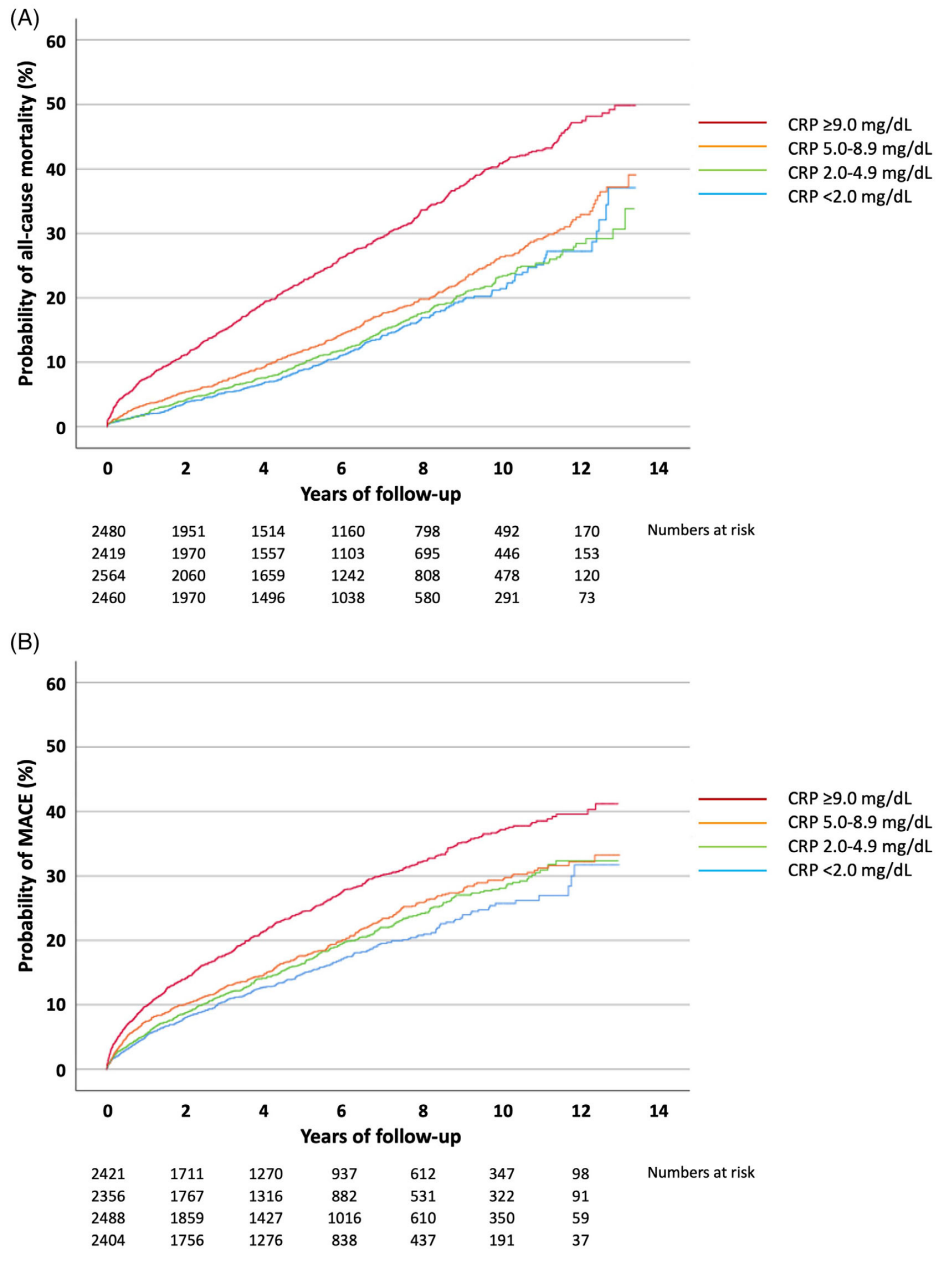
Cumulative probability of (A) all‐cause mortality and )B) major cardiovascular events in patients with myocardial infarction with nonobstructive coronary arteries (MINOCA) in relation to C‐reactive protein (CRP) quartiles

During a median follow‐up of 5.3 (2.3–8.6) years, 1854 (18.7%) MINOCA patients died. Totally 1939 (20.1%) MINOCA patients suffered a MACE during a median follow‐up of 4.2 (1.5–7.6) years. Increasing CRP quartiles were significantly associated with both outcomes with continuously diverging Kaplan–Meier curves, in particular for patients in the top vs. the lower three CRP quartiles (Table 1; Figures 1(A),(B)). The associations of CRP quartiles with MACE were mainly driven by higher rates of mortality and hospitalizations for heart failure (Table 1). The multivariable‐adjusted HR of CRP (ln) were 1.21 (95% CI 1.17–1.26) regarding all‐cause mortality and 1.08 (95% CI 1.04–1.12) regarding MACE (Table 3). Further adjustments for left‐ventricular ejection fraction or hs‐cTnT (ln) did not alter these associations. The corresponding HR in MI‐CAD patients were 1.22 (95% CI 1.20–1.23) regarding all‐cause mortality and 1.17 (95% CI 1.16–1.18) regarding MACE. The interactions of the type of MI (MINOCA vs. MI‐CAD) on the associations of CRP (ln) were non‐significant regarding all‐cause mortality but reached significance regarding MACE.

**TABLE 3:** Association of CRP (ln) with adverse outcomes

	MINOCA			MI‐CAD			
	*n*	HR (95% CI)	p value	*n*	HR (95% CI)	p value	p int.
Model 1							
All‐cause mortality	9709	1.22 (1.17–1.26)	<.001	96 221	1.22 (1.21–1.23)	<.001	0.904
MACE	9469	1.08 (1.04–1.12)	<.001	93 813	1.17 (1.16–1.18)	<.001	<.001
Model 2							
All‐cause mortality	7971	1.21 (1.16–1.26)	<.001	81 353	1.18 (1.16–1.19)	<.001	0.329
MACE	7761	1.09 (1.04–1.13)	<.001	79 116	1.12 (1.11–1.13)	<.001	.019
Model 3							
All‐cause mortality	4095	1.23 (1.15–1.32)	<.001	42 971	1.24 (1.21–1.26)	<.001	0.764
MACE	3926	1.09 (1.02–1.16)	.013	41 142	1.18 (1.16–1.20)	<.001	.021

Abbreviations: CI, confidence interval; HR, hazard ratio; MACE, major adverse cardiovascular event.

*Note*: Model 1: adjusted for sex, age, current smoking, hypertension, diabetes, hyperlipidemia, estimated glomerular filtration rate, previous heart failure, previous stroke, peripheral vascular disease, chronic obstructive pulmonary disease, dementia, previous or present cancer, ST‐segment changes, atrial fibrillation, in‐hospital coronary revascularization (if appropriate), hospital and admission year. Model 2: adjusted as model 1 with replacement of heart failure by left‐ventricular ejection fraction. Model 3: adjusted as model 1 with additional adjustment for high‐sensitivity cardiac troponin T (ln). p _int_.: p value referring to the interaction of the type of MI (MINOCA vs. MI‐CAD) on the association of CRP (ln) with adverse outcome.

## 4 DISCUSSION

In the present analysis investigating a large cohort of MINOCA patients, CRP levels yielded moderate but independent prognostic information. Increases of one standard deviation in ln‐transformed CRP levels were associated with 21–23% risk increases regarding all‐cause mortality and 8%–9% risk increases regarding MACE in models considering cardiovascular risk factors, comorbidities and estimates of myocardial damage.

However, contrasting to our hypothesis, CRP levels did not provide unique prognostic information compared to patients with MI‐CAD. The prognostic value of CRP was similar for MINOCA and MI‐CAD patients regarding all‐cause mortality, and its magnitude regarding MACE was relatively lower in MINOCA patients compared to those with MI‐CAD. This is likely explained by the fact that CRP levels in MI at least in part reflect an acute‐phase reaction resulting from myocardial necrosis. Patients with MI‐CAD from our cohort suffered greater myocardial damage compared to those with MINOCA, as demonstrated by more than 3‐fold higher hs‐cTnT levels and more severely depressed left‐ventricular ejection fraction, strengthening the association between CRP and MACE. Still, CRP provided similar prognostic information regarding all‐cause mortality in both MI types. The predictive value of CRP in MINOCA should thus, not be neglected.

Our findings corroborate with data reported by Ciliberti et al. from a small MINOCA cohort demonstrating that CRP predicted MACE independently of the extent of coronary atheromatosis.^10^ This raises the question whether CRP levels might represent other disease mechanisms compared to those being relevant in MI‐CAD. However, the multiple linear regressions did not reveal a clear MINOCA‐specific pattern of contributing entities. Accordingly, the null hypothesis of CRP levels providing distinct clinical information in MINOCA was not rejected. In this context, it should be acknowledged that CRP levels do not necessarily reflect the proinflammatory disposition that may exist in MINOCA patients. Indirect support comes from a recent analysis from our group investigating a large array of circulating biomarkers in a stable post‐MINOCA cohort.^7^ Here, various pro‐ and anti‐inflammatory biomarkers other than CRP were found to discriminate MINOCA from both MI‐CAD and healthy controls.

The greatest clinical value of CRP, besides prognostication, appears to lie in the distinction of true MINOCA from myocarditis mimicking MI. While cardiac magnetic resonance imaging is regarded as gold standard in this regard,^1^ CRP levels may guide early diagnostic work‐up. CRP >10 mg/dl for example, strongly suggested the presence of myocarditis in a pooled cohort of 556 patients regarded having MINOCA.^11^ Likewise, CRP <5 mg/dl was shown to be associated with the presence of findings consistent with small‐vessel obstruction in another study investigating 135 patients with presumed MINOCA.^12^ It can sometimes also be difficult to distinguish MINOCA from Takotsubo cardiomyopathy. Even in this regard, CRP may provide a clue. Takotsubo cardiomyopathy is characterized by a myocardial and systemic inflammatory reaction^13^ associated with an elevation in CRP levels rather seen in ST‐segment elevation MI^14^ than in MINOCA.

Our study has several strengths. We assessed a large cohort of MINOCA patients with information on various clinically relevant outcomes. Because of the unique Swedish personal identification number and mandatory health registries, we have complete information on outcome in all patients over several years of follow‐up. There are also some limitations that need to be considered. This was a retrospective investigation from a registry with inherent selection bias. Although all hospitals participating in SWEDEHEART are annually monitored, the data cannot be of the same quality as in a prospective observational study. However, the agreement between the information entered in the registry and the medical records is around 96%.^15^ We lack detailed information on the assays used for CRP measurements. We would not regard this as a major shortcoming since guidelines recommend the use of uniform prognostic CRP thresholds despite inter‐assay variations in detection ranges.^16^ We are unable to comment on the potential associations of the severity of atheromatosis with CRP levels in MINOCA since the data available in SWEDEHEART do not allow for a subclassification of patients with coronary stenosis <50%. Moreover, we lack information on intravascular imaging which might have identified high‐risk plaque morphologies contributing to higher CRP levels in some MINOCA patients. We do not have data on the results of examinations performed after the hospitalization period. Thus, some MINOCA patients may have subsequently been diagnosed with myocarditis, Takotsubo cardiomyopathy or any other less common diagnosis on the basis of post‐discharge cardiac magnetic resonance imaging. CRP is an unspecific biomarker of inflammatory activity. SWEDEHEART provides no information on infectious or non‐cardiovascular inflammatory disorders that may have contributed to higher CRP levels. Finally, data on MACE were only available until December 2017.

Our results in conclusion, demonstrate that CRP is independently, albeit moderately associated with adverse outcome in MINOCA. Contrasting to our primary hypothesis, we found no evidence indicating that acute MINOCA might be characterized by a specific inflammatory pattern, as reflected by CRP levels.

## CONFLICT OF INTEREST

None of the authors reported conflicts of interest relevant to this study.

## Supporting information


**Table S1** Clinical characteristics and outcome in patients with MINOCA and MI‐CAD.
**Table S2**. Predictors of CRP levels in patients with A) MINOCA and B) MI‐CAD.

## Data Availability

The data used in this study originates from the SWEDEHEART registry and contains sensitive patient information. The dataset analyzed in this study is not publicly available due to Swedish patient privacy and secrecy laws regulating access to SWEDEHEART, and due to ethical restrictions regarding the current analysis from the TOTAL‐AMI project (Regional Ethical Review Board in Stockholm; reference number 2012/60‐31/2). We hereby confirm that other researchers are able to access the data at Uppsala Clinical Research Center upon reasonable request and under the provision that the data is accessed onsite and does not leave Uppsala University. This request can be sent to info@ucr.uu.se.
