# Supplementary material for: Clinical and prognostic implications of C‐reactive protein levels in myocardial infarction with nonobstructive coronary arteries
Source: Clin Cardiol. 2021 May 25;44(7):1019–27. doi: 10.1002/clc.23651 (PMC8259160; doi:10.1002/clc.23651)
Supplement: Supplementary file 1 — Table S1 Clinical characteristics and outcome in patients with MINOCA and MI‐CAD. Table S2. Predictors of CRP levels in patients with A) MINOCA and B) MI‐CAD. [file CLC-44-1019-s001.docx]

**Supplemental Material.**

**Supplemental Table 1. Clinical characteristics and outcome in patients with MINOCA and MI-CAD.**

|  | **MINOCA**  **(n=9916)** | **MI-CAD (n=97,970)** | **p-value** | **Missing values** |
| --- | --- | --- | --- | --- |
|  |  |  |  |  |
|  |  |  |  |  |
| **Risk factors** |  |  |  |  |
| Age (years) | 67 (58-75) | 68 (59-76) | <0.001 | - |
| Males | 3662 (36.9%) | 68,783 (63.8%) | <0.001 | - |
| Current smoking | 1797 (18.1%) | 25,588 (26.1%) | <0.001 | 117 (0.1%) |
| Hypertension | 4414 (44.5%) | 44,286 (45.2%) | 0.186 | 73 (0.1%) |
| Diabetes | 1167 (11.8%) | 16,134 (16.5%) | <0.001 | 1 (0%) |
| Hyperlipidemia | 1851 (18.7%) | 16,414 (16.8%) | <0.001 | 6 (0%) |
| BMI (kg/m^2^) | 26.1 (23.4-29.4) | 26.6 (24.2-29.4) | <0.001 | 13,114 (12.2%) |
| eGFR (mL/min/1.73m^2^) | 82.0 (66.0-93.2) | 81.5 (65.3-93.1) | 0.017 | 216 (0.2%) |
|  |  |  |  |  |
| **CV history** |  |  |  |  |
| Heart failure | 293 (3.0%) | 1801 (1.8%) | <0.001 | 7 (0%) |
| Previous stroke | 470 (4.8%) | 5025 (5.2%) | 0.090 | 787 (0.7%) |
| PAD | 205 (2.1%) | 2774 (2.8%) | <0.001 | - |
| COPD | 914 (9.2%) | 4916 (5.0%) | <0.001 | - |
| Previous/present cancer | 226 (2.3%) | 1784 (1.8%) | 0.002 | - |
| Dementia | 18 (0.2%) | 261 (0.3%) | 0.125 | - |
|  |  |  |  |  |
| **ECG findings** |  |  |  |  |
| Sinus rhythm | 8740 (88.3%) | 89,431 (91.5%) | <0.001 | 198 (0.2%) |
| Atrial fibrillation | 904 (9.1%) | 6016 (6.2%) | <0.001 | 198 (0.2%) |
| ST-elevation | 1344 (13.6%) | 41,038 (42.0%) | <0.001 | 309 (0.3)% |
| ST-depression | 1657 (16.8%) | 19,193 (19.6%) | <0.001 | 309 (0.3)% |
|  |  |  |  |  |
| **Medications at admission** | |  |  |  |
| Aspirin | 1865 (18.8%) | 17,588 (18.0%) | 0.036 | 6 (0%) |
| P2Y12 blockers | 243 (2.5%) | 2315 (2.4%) | 0.582 | 6 (0%) |
| Oral anticoagulants | 549 (5.5%) | 2944 (3.0%) | <0.001 | 6 (0%) |
| Betablockers | 2531 (25.5%) | 21,784 (22.2%) | <0.001 | 6 (0%) |
| RAAS-inhibitors | 2980 (30.1%) | 25,734 (26.3%) | <0.001 | 6 (0%) |
| CCB | 1241 (12.5%) | 15,522 (15.8%) | <0.001 | 6 (0%) |
| Statins | 1794 (18.1%) | 15,973 (16.3%) | <0.001 | 6 (0%) |
|  |  |  |  |  |
| **Examination results** |  |  |  |  |
| hs-cTnT (ng/L)* | 187 (72-483) | 669 (167-2470) | <0.001 | - |
| Echocardiographic findings † | |  |  |  |
| LVEF ≥0.50 | 6047 (74.3%) | 50,255 (60.7%) | <0.001 | - |
| LVEF 0.40-0.49 | 1150 (14.1%) | 18,367 (22.2%) |  |  |
| LVEF 0.30-0.39 | 655 (8.0%) | 10,263 (12.4%) |  |  |
| LVEF <0.30 | 287 (3.5%) | 3927 (4.7%) |  |  |
|  |  |  |  |  |
| **Medications at discharge** ‡ | |  |  |  |
| Aspirin | 8604 (87.3%) | 91,551 (95.9%) | <0.001 | - |
| P2Y12 blockers | 6606 (67.0%) | 84,584 (88.6%) | <0.001 | - |
| Oral anticoagulants | 929 (9.4%) | 5444 (5.7%) | <0.001 | 5 (0%) |
| Betablockers | 7841 (79.6%) | 86,161 (90.2%) | <0.001 | - |
| RAAS-inhibitors | 6346 (64.4%) | 75,200 (78.8%) | <0.001 | - |
| CCB | 1645 (16.7%) | 12,423 (13.0%) | <0.001 | 1 (0%) |
| Statins | 8277 (84.0%) | 90,477 (94.8%) | <0.001 | - |
|  |  |  |  |  |
|  | **MINOCA**  **(n=9916)** | **MI-CAD (n=97,970)** | **p-value** | **Missing values** |
|  |  |  |  |  |
|  |  |  |  |  |
| **Crude event rates ¶** |  |  |  |  |
| All-cause mortality | 1854 (18.7%) | 21,950 (22.4%) | <0.001 | - |
| CV mortality | 637 (6.6%) | 9721 (10.2%) | <0.001 | - |
| MI | 667 (6.9%) | 8471 (8.9%) | <0.001 | - |
| Heart failure | 645 (6.7%) | 7041 (7.4%) | 0.011 | - |
| Stroke | 455 (4.7%) | 3970 (4.2%) | 0.011 | - |
| MACE | 1939 (20.1%) | 22,993 (24.1%) | <0.001 | - |
|  |  |  |  |  |

Data given as numbers (with percentages) or medians (with interquartile ranges).

* n=47,675.

† Echocardiography was performed in 92,465 patients (85.7%). Data on LVEF was available in 90,951 of these patients.

‡ Assessed in in-hospital survivors: n=105,323.

¶ Follow-up data regarding all-cause mortality were available in all patients (n=9916) until May 2018, and regarding MACE and its individual components in 105,165 patients until December 2017.

BMI: body mass index; eGFR: estimated glomerular filtration rate; CV: cardiovascular; PAD: peripheral artery disease; COPD: chronic obstructive pulmonary disease; RAAS: renin-angiotensin-aldosterone system; CCB: calcium channel blockers; hs-cTnT: high-sensitivity cardiac troponin T; LVEF: left-ventricular ejection fraction; MI: myocardial infarction; CV: cardiovascular; MACE: major adverse cardiovascular events.

**Supplemental Table 2. Predictors of CRP levels in patients with A) MINOCA and B) MI-CAD.**

| **A)** | **Model 1 (n=9716)** | | | **Model 2 (n=8061)** | | |  |
| --- | --- | --- | --- | --- | --- | --- | --- |
|  |  | | |  | | |  |
|  |  | | |  | | |  |
|  | **B (95% CI)** | **β** | **p value** | **B (95% CI)** | **β** | **p value** | |
|  |  |  |  |  |  |  | |
|  |  |  |  |  |  |  | |
| Age (10 years) | 0.045 (0.019 to 0.072) | 0.042 | <0.001 | 0.027 (-0.002 to 0.056) | 0.025 | 0.070 | |
| Male sex | 0.050 (-0.001 to 0.102) | 0.019 | 0.056 | 0.052 (-0.004 to 0.108) | 0.020 | 0.070 | |
| Current smoking | 0.197 (0.131 to 0.262) | 0.061 | <0.001 | 0.167 (0.096 to 0.239) | 0.052 | <0.001 | |
| Hypertension | -0.002 (-0.066 to 0.063) | -0.001 | 0.962 | 0.016 (-0.055 to 0.087) | 0.006 | 0.655 | |
| Diabetes | 0.238 (0.159 to 0.317) | 0.061 | <0.001 | 0.204 (0.117 to 0.292) | 0.053 | <0.001 | |
| Hyperlipidemia | 0.093 (-0.225 to 0.410) | 0.029 | 0.567 | 0.120 (-0.240 to 0.480) | 0.037 | 0.514 | |
| eGFR (ln) | -0.005 (-0.006 to -0.003) | -0.075 | <0.001 | -0.003 (-0.005 to -0.002) | -0.053 | <0.001 | |
| Heart failure | 0.180 (0.033 to 0.327) | 0.025 | 0.016 | - | - | - | |
| Previous stroke | 0.005 (-0.113 to 0.123) | 0.001 | 0.928 | -0.033 (-0.162 to 0.096) | -0.006 | 0.615 | |
| PAD | 0.500 (0.325 to 0.675) | 0.056 | <0.001 | 0.603 (0.411 to 0.795) | 0.068 | <0.001 | |
| COPD | 0.447 (0.361 to 0.532) | 0.103 | <0.001 | 0.392 (0.298 to 0.486) | 0.090 | <0.001 | |
| Cancer | 0.248 (0.085 to 0.410) | 0.030 | 0.003 | 0.264 (0.078 to 0.451) | 0.030 | 0.006 | |
| Dementia | -0.313 (-0.891 to 0.265) | -0.010 | 0.288 | -0.073 (-0.758 to 0.612) | -0.002 | 0.835 | |
| Atrial fibrillation | 0.180 (0.090 to 0.271) | 0.042 | <0.001 | 0.177 (0.077 to 0.277) | 0.040 | 0.001 | |
| LVEF | - | - | - | 0.183 (0.148 to 0.217) | 0.115 | <0.001 | |
|  |  |  |  |  |  |  | |

| **B)** | **Model 1 (n=96,224)** | | | **Model 2 (n=81,654)** | | |
| --- | --- | --- | --- | --- | --- | --- |
|  |  | | |  | | |
|  |  |  |  |  |  |  |
|  | **B (95% CI)** | **β** | **p value** | **B (95% CI)** | **β** | **p value** |
|  |  |  |  |  |  |  |
|  |  |  |  |  |  |  |
| Age (10 years) | 0.015 (0.006 to 0.024) | 0.014 | 0.001 | -0.004 (-0.014 to 0.005) | -0.004 | 0.365 |
| Male sex | -0.039 (-0.057 to -0.021) | -0.014 | <0.001 | -0.049 (-0.068 to -0.029) | -0.017 | <0.001 |
| Current smoking | 0.193 (0.173 to 0.212) | 0.066 | <0.001 | 0.162 (0.142 to 0.183) | 0.055 | <0.001 |
| Hypertension | 0.015 (-0.006 to 0.036) | 0.006 | 0.170 | 0.027 (0.004 to 0.050) | 0.010 | 0.022 |
| Diabetes | 0.252 (0.229 to 0.275) | 0.072 | <0.001 | 0.226 (0.202 to 0.251) | 0.065 | <0.001 |
| Hyperlipidemia | -0.180 (-0.298 to -0.061) | -0.052 | 0.003 | -0.062 (-0.190 to 0.065) | -0.018 | 0.388 |
| eGFR | -0.009 (-0.009 to -0.008) | -0.139 | <0.001 | -0.007 (-0.008 to -0.007) | -0.114 | <0.001 |
| Heart failure | 0.216 (0.155 to 0.276) | 0.022 | <0.001 | - | - | - |
| Previous stroke | 0.108 (0.070 to 0.146) | 0.019 | <0.001 | 0.077 (0.035 to 0.118) | 0.013 | <0.001 |
| PAD | 0.297 (0.248 to 0.346) | 0.038 | <0.001 | 0.263 (0.210 to 0.316) | 0.033 | <0.001 |
| COPD | 0.338 (0.301 to 0.374) | 0.057 | <0.001 | 0.314 (0.274 to 0.353) | 0.053 | <0.001 |
| Cancer | 0.254 (0.195 to 0.314) | 0.026 | <0.001 | 0.248 (0.184 to 0.312) | 0.025 | <0.001 |
| Dementia | -0.005 (-0.159 to 0.148) | 0.000 | 0.944 | -0.090 (-0.268 to 0.088) | -0.003 | 0.320 |
| Atrial fibrillation | 0.273 (0.238 to 0.309) | 0.051 | <0.001 | 0.209 (0.171 to 0.248) | 0.038 | <0.001 |
| LVEF | - | - | - | 0.282 (0.272 to 0.291) | 0.191 | <0.001 |
|  |  |  |  |  |  |  |

Model 1: adjusted for all assessed variables including hospital, admission year and medications at admission as listed in Table 1.

Model 2: adjusted as model 1 with replacement of heart failure by left-ventricular ejection fraction, categorized in as ≥0.50, 0.40-0.49, 0.30-0.39 and <0.30.

CI: confidence interval; eGFR: estimated glomerular filtration rate; PAD: peripheral artery disease; COPD: chronic obstructive pulmonary disease; LVEF: left-ventricular ejection fraction.
